# Coupling of HIV-1 Antigen to the Selective Autophagy Receptor SQSTM1/p62 Promotes T-Cell-Mediated Immunity

**DOI:** 10.3389/fimmu.2016.00167

**Published:** 2016-05-10

**Authors:** Aram Nikolai Andersen, Ole Jørgen Landsverk, Anne Simonsen, Bjarne Bogen, Alexandre Corthay, Inger Øynebråten

**Affiliations:** ^1^Tumor Immunology Group, Department of Pathology, Rikshospitalet, University of Oslo and Oslo University Hospital, Oslo, Norway; ^2^Department of Immunology, Rikshospitalet, University of Oslo and Oslo University Hospital, Oslo, Norway; ^3^Centre for Immune Regulation, University of Oslo, Oslo, Norway; ^4^LIIPAT, Department of Pathology, Rikshospitalet, Oslo University Hospital, Oslo, Norway; ^5^Department of Molecular Medicine, Institute of Basic Medical Sciences, University of Oslo, Oslo, Norway; ^6^K. G. Jebsen Centre for Influenza Research, University of Oslo, Oslo, Norway

**Keywords:** vaccine, T cell responses, p62/SQSTM1, autophagy, HIV-1 gagp24 antigen

## Abstract

Vaccines aiming to promote T-cell-mediated immune responses have so far showed limited efficacy, and there is a need for novel strategies. Studies indicate that autophagy plays an inherent role in antigen processing and presentation for CD4^+^ and CD8^+^ T cells. Here, we report a novel vaccine strategy based on fusion of antigen to the selective autophagy receptor sequestosome 1 (SQSTM1)/p62. We hypothesized that redirection of vaccine antigen from proteasomal degradation into the autophagy pathway would increase the generation of antigen-specific T cells. A hybrid vaccine construct was designed in which the antigen is fused to the C-terminus of p62, a signaling hub, and a receptor that naturally delivers ubiquitinated cargo for autophagic degradation. Fusion of the human immunodeficiency virus-1 antigen Gagp24 to p62 resulted in efficient antigen delivery into the autophagy pathway. Intradermal immunization of mice revealed that, in comparison to Gagp24 delivered alone, fusion to p62 enhanced the number of Gagp24-specific interferon-γ-producing T cells, including CD8^+^ T cells. The strategy may also have the potential to modulate the antigenic peptide repertoire. Because p62 and autophagy are highly conserved between species, we anticipate this strategy to be a candidate for the development of T-cell-based vaccines in humans.

## Introduction

T-cell-based vaccines have the potential to confer protection against cancer and pathogens. CD4^+^ T cells may lead to the killing of target cells in an indirect or direct manner (1–3), and they are crucial for the generation of long-lived, memory CD8^+^ T cells (4–6). CD8^+^ T cells kill in a direct manner, and several studies have proven their ability to fight pathogens and reject cancer (7, 8). However, vaccines aiming to promote T-cell-mediated immune responses have so far showed limited efficacy. Thus, development of technologies that can improve T cell responses, in particular CD8^+^ T cell responses, is a major goal in vaccine design and immunotherapy.

Antigen-presenting cells (APCs) present peptides on major histocompatibility complex (MHC) class I molecules to CD8^+^ T cells and on MHC class II molecules to CD4^+^ T cells. For presentation on MHC class II molecules, extracellularly or intracellularly derived antigenic protein fragments are generated in endolysosomal compartments and peptides are loaded on MHC class II molecules in late endosomes/MHC class II molecule-containing compartments (MIIC) (9). The conventional pathway for MHC class I antigen presentation includes proteasomal degradation in the cytosol of APC and transporter associated with antigen processing (TAP)-mediated delivery of peptides into the lumen of endoplasmic reticulum. There, peptides are loaded on MHC class I before transport of the peptide/MHC complex to the cell surface (10). The mechanism, whereby *extracellular* antigens are presented by APCs on MHC class I, is termed cross-presentation. The specific mechanisms are still debated but may involve export of endocytosed antigen into the cytosol for further degradation and loading of peptides on MHC class I molecules in the endoplasmic reticulum or in endosomes (10–12).

A number of studies have pointed to the role of autophagy and autophagy proteins in antigen delivery and adaptive immunity (12–19). Autophagy is an intracellular degradation system that delivers constituents to the lysosome. During macroautophagy (hereafter autophagy) cytosolic proteins, damaged and superfluous organelles, as well as invasive microorganisms are sequestered into a double-membrane vesicle called autophagosome, which undergoes fusion with endolysosomal compartments (20). Thus, the autophagic pathway facilitates presentation of cytoplasm-derived antigens on MHC class II molecules (13, 21–25).

Several studies also suggest a role for autophagy in MHC class I-restricted antigen presentation and engagement of CD8^+^ T cells. For example, it has been shown that autophagy in the APC can enhance MHC class I-restricted presentation of endogenous, cytoplasm-derived, and cross-presented antigens (17, 26, 27). In addition, autophagy in the antigen donor cell has been implicated in enhanced cross-priming of CD8^+^ T cells and improved protection against cancer in mouse models (17, 28–31). Finally, the use of enriched autophagic vesicles as source of antigen suggested that such vesicles can contribute to cross-presentation of antigens to CD8^+^ T cells (28, 30, 31). Taken together, several studies indicate that autophagy plays an inherent role in antigen processing and presentation for CD4^+^ and CD8^+^ T cell activation.

Aiming to develop a vaccine with enhanced T cell priming capacity, we hypothesized that targeting of vaccine antigen to the autophagy pathway would promote antigen-specific T cell responses and mimic how APCs naturally encounter an infection or a cancerous cell. In order to target vaccine antigen to the autophagy pathway, we utilized sequestosome 1 (SQSTM1)/p62. We chose p62 because it is a selective autophagy receptor that naturally delivers a broad range of ubiquitinated cargo into the autophagy pathway, and we anticipated that it would tolerate fusion with various antigens (32–35). Furthermore, p62 mediates NF-κB-activation, which could be beneficial for vaccine responses (36–38).

In this study, p62 was fused to the human immunodeficiency virus-1 (HIV-1) antigen Gagp24. Although many studies have suggested that CD8^+^ T cell responses against Gagp24 or its precursor Gag are important to maintain a low viral load and delay the onset of AIDS (39–43), conventional Gag-based vaccine strategies have failed to induce protective T cell responses. Thus, we wanted to explore the potential of an autophagy receptor and hypothesized that fusion of p62 to HIV-1 Gagp24 would rescue the antigen from rapid proteasomal degradation and potentiate antigen-specific T cell responses.

## Materials and Methods

### Reagents

The following reagent was obtained through the Centre for AIDS Reagent, Division of Virology, NIBSC, UK: HIV-1 Con B Gag peptides (ARP7111) from the Division of AIDS, NIAID, USA. The peptides comprise HIV-1 HXB2 Gagp24 and are 15-mers with 11-amino acid overlaps between sequential peptides.

### Cell Lines and Cultivation

HEK293 cells stably transfected with EGFP–LC3, denoted EGFP–LC3^+^ 293 cells, were kindly provided by Sharon Tooze (Francis Crick Institute, London, UK) (44). EGPF-LC3^+^ 293 cells and HEK293T cells were cultured in RPMI 1640 (Life Technologies, Carlsbad, CA, USA) supplemented with 10% heat-inactivated fetal bovine serum (FBS) (Biochrom AG, Berlin, Germany), 0.1 μM non-essential amino acids (Lonza, Basel, Switzerland), 1 mM sodium pyruvate (Lonza), 50 μM monothioglycerol (Sigma-Aldrich, St. Louis, MO, USA), and 40 mg/ml gensumycin (Sanofi-Aventis Norge AS, Lysaker, Norway) (herein, referred to as complete RPMI). For live-cell confocal microscopy, RPMI 1640 without phenol red (Life Technologies) was used. All mammalian cells were grown at 37°C and 5% CO_2_.

### Mice

Female C57BL/6 and BALB/c mice were purchased from Taconic (Ry, Denmark). All mice were acclimatized to the animal research facility Department of Comparative Medicine, Oslo University Hospital, Rikshospitalet and were included in experiments when they were 6–10 weeks of age. The study was approved by the National Committee for Animal Experiments, Norway (permit number: Id-4420), and the experiments were performed in accordance with the approved guidelines and regulations.

### Plasmids and Design of Constructs

pBudCE4.1 (Life Technologies) was used as an expression vector to retain the possibility of dual expression of genes in the future. For simple and versatile subcloning of all constructs in this study, we modified pBudCE4.1 and inserted a fragment containing the restriction sites 5′-*Hin*dIII–*Sal*I–SfiI_1_–SfiI_2_–*Xba*I in the multiple cloning site downstream of the CMV promoter. The cDNA sequence of HIV-1 Gagp24, isolate BH10 (GenBank accession number M15654.1 and nucleotide 508–1200), was ordered from GenScript (Piscataway, NJ, USA) with *Sfi*I restriction sites in the 5- and 3-prime end (SfiI_1_: 5′ GGCC*TCAGC*GGCC TG- and SfiI_2_: GGCC*TGCA*GGGCC-3′). Human p62/SQSTM1 cDNA was retrieved by PCR from a pDEST-EGFP vector (Terje Johansen, Institute of Medical Biology, University of Tromsø, Norway) by PCR and primers, including overhangs for *Hin*dIII (P_fwd_: 5′-AAATTT*AAGCTT*GATGGCGTCGCTCACCGTGAAG G-3′) and *Sal*I (P_rev_: 5′-AAATTT*GTCGAC*CTCAACGGCGGGGGATGCTTTGAATA-3′). cDNA encoding mCherry was amplified by PCR with primers, including overhangs for SfiI_1_ (P_fwd_: 5′-AAATTT*GGCCTCAGCGGCC* TGGTGAGCAAGGGCGAGGAGGAT-3′) and SfiI_2_ (P_rev_: 5′-AAATTT*GGCCCTGCAGGCC*TTACTTGTACAG CTCGTCCATGCCG-3′). To conjugate Gagp24 with mCherry, Gagp24 cDNA was amplified by PCR with primers, including overhangs for *Hin*dIII (P_fwd_: 5′-AAATTT*AAGCTT*GATGCCCATCGTGCAGAACATCCAG-3′) and *Sal*I (P_rev_: 5′-AAATTT*GTCGAC*CCCAGCACTCTAGCCTTATGGCC-3′). Plasmid encoding mCherry only was generated by PCR and primers that contained overhangs for *Hin*dIII (P_fwd_: 5′-AAATTTAAGCTTGATGGTGAGCAAGGGCGAGGAG-3′) and *Xba*I (P_rev_: 5′-AAATTTTCTAGACTT GTACAGCTCGTCCATGCCG-3′).

### DNA Transfection for Transient Protein Expression

All DNA plasmids were transfected into cells using Lipofectamine LTX with PLUS-reagent (Life Technologies). Cells were seeded at a density of 1000 cells/mm^2^ in 24-well plates or 8-well Nunc^®^ Lab-Tek^®^ II Chambered Coverglass (Sigma-Aldrich). Twenty-four hours after seeding, the cells were transfected with 0.5 μg plasmid per well in 24-well plates, according to the manufacturer’s instructions (Life Technologies). Co-transfections of two plasmids were performed by using 0.25 μg DNA of each plasmid.

### Western Blotting

For assessment of intracellular protein levels, cell cultures were washed in phosphate-buffered saline (PBS) (Lonza), and then lysed in CytoBuster™ protein extraction reagent (EMD Millipore, Billerica, MA, USA) on ice for 15 min. The lysates were cleared by 2000 × *g* centrifugation for 15 min at 4°C. Proteins were denatured by mixing 20 μl lysate with 4 μl sample loading solution [12% sodium dodecyl sulfate (SDS) with 3 mM Tris pH 6.8 and 0.05% bromophenol blue], followed by incubation at 95°C for 5 min. The samples were run on a 4–12% Novex Tris-Glycine Gel (Life Technologies) and blotted onto an Immun-Blot™ PVDF membrane (Bio-Rad Laboratories, Hercules, CA, USA) in PBS with 0.1% tween. The membranes were blocked with 4% ECL Advance™ Blocking Reagent (GE Healthcare, Pittsburgh, PA, USA) at room temperature for 1.5 h. Afterward, the membranes were incubated with a mouse monoclonal anti-HIV-1-p24 antibody (0.5 μg/ml) (Abcam, Cambridge, UK) or a mouse monoclonal anti-β-tubulin antibody (0.5 μg/ml) (Abcam) at 4°C for 12 h. Next, the membrane was incubated with a horseradish peroxidase-conjugated rabbit anti-mouse IgG antibody (1 μg/ml) (Life Technologies) at room temperature for 1.5 h. Bound antibodies were visualized using ECL Advance™ Western blotting detection kit (GE Healthcare, Little Chalfont, UK). Image acquisition was performed using the ChemiDoc MP system (Bio-Rad Laboratories), and the bands were analyzed by ImageLab software version 4.1 (Bio-Rad Laboratories).

### Immunostaining

Cell cultures were washed once in PBS and fixed in 4% formaldehyde solution at 4°C for 45 min. To remove the fixative, cells were washed in PBS four times. Next, PBS containing 5% albumin and 0.05% saponin was used to reduce unspecific binding and to permeabilize the cells before immunostaining with a mouse monoclonal anti-HIV-1-p24 antibody (0.5 μg/ml) (Abcam) for 2 h at room temperature, followed by three washes and a secondary staining for 1 h using goat anti-mouse conjugated with Alexa Fluor 568 (Abcam). Finally, the cells were washed and mounted using ProLong Gold Antifade Mountant with DAPI (Life Technologies).

### Microscopy, Sampling, and Quantitation

Confocal micrographs were taken with Olympus FluoView™ using an inverted FV1000 confocal microscope (Olympus America, Center Valley, PA, USA). The diode laser (405 nm) was used to excite DAPI, the argon laser (488 nm) to excite EGFP-conjugates, and the helium–neon laser (543 nm) was used to excite mCherry and Alexa Fluor 568. All imaging experiments were performed three times. Images were systematically acquired throughout each slide in a uniform fashion. To quantitate the number of fluorescent puncta or vesicles and co-localization per cell, all images were counted. Representative images were chosen for publishing. Image editing was conducted in Image J and Adobe Illustrator CS5.

### DNA Vaccination

For all *in vivo* experiments, DNA plasmids were prepared using large-scale EndoFree Qiagen kit (Qiagen, Hilden, Germany) and diluted to concentration 0.5 μg/μl in 0.9% NaCl. Mice were anesthetized, and their lower back was shaved before 25 μl plasmid solution was injected into the dermis on each flank (i.e., 12.5 μg DNA × 2 per mouse). Immediately afterward, the injection site was exposed to electroporation using DermaVax (BTX Harvard Apparatus, Holliston, MA, USA), applying two pulses of 450 V/cm × 2.5 μs and eight pulses of 110 V/cm × 8.1 ms *via* a needle array electrode.

### Interferon-γ-ELISpot

Spleens from mice were crushed by a steel mesh to form single-cell suspensions. In order to lyse the erythrocytes, the cell suspensions were treated with 140 mM NH_4_Cl in Tris-buffer (pH = 7.2) for 5–10 min. Gagp24-reactive cellular immune responses in vaccinated mice were assessed by pre-coated interferon-γ (IFNγ)-ELISpot plates, according to the manufacturer’s protocol (Mabtech, Nacka Strand, Sweden). Splenocytes in RPMI 1640 with 10% FBS and gensumycin were seeded at cell density 1 × 10^6^, 5 × 10^5^, and 2.5 × 10^5^ in volume 100 μl per well in duplicates and restimulated with peptides in concentration 4 μg/μl for 26–28 h at 37°C. The peptides comprised the entire Gagp24 and were 15-mers with 11-amino acid overlaps between sequential peptides. The peptides were divided in pools 1–5, as described by Trumpfheller et al., and each pool consisted of 9–12 peptides (45). Pool 1 consisted of peptides spanning amino acid 125–183 of Gag polyprotein precursor; pool 2, amino acid 173–231; pool 3, amino acid 221–279; pool 4, amino acid 269–327; and pool 5, amino acid 317–363. In addition, the MHC class I-restricted, H-2 Kd-binding peptide AMQMLKETI (amino acid 197–205) (GenScript) was used for restimulation of CD8^+^ T cells (46). Splenocytes cultivated in medium only and splenocytes from mice that had received NaCl before electroporation were used as negative controls. The number of IFNγ^+^ spots was determined by CTL ELISPOT reader (CTL Europe GmbH, Bonn, Germany) in “Smart Count” mode. Before enumeration of the samples, the software was trained to set a threshold for spots that should be considered as positive, by analyzing several wells of negative controls as well as strongly positive samples. Occasionally, negative control wells contained a few spots (1–13), which were not substracted from the samples. All counted spots for the p62–Gagp24 and Gagp24 group of mice are presented.

### Statistical Analyses

Statistical analyses were performed using GraphPad Prism version 4. Differences between treatment groups were analyzed by using two-tailed *t*-test or Mann–Whitney test. *P*-values ≤0.05 were considered significant.

## Results

### Design of SQSTM1/p62-Containing Vaccine Construct

**Figure 1:**
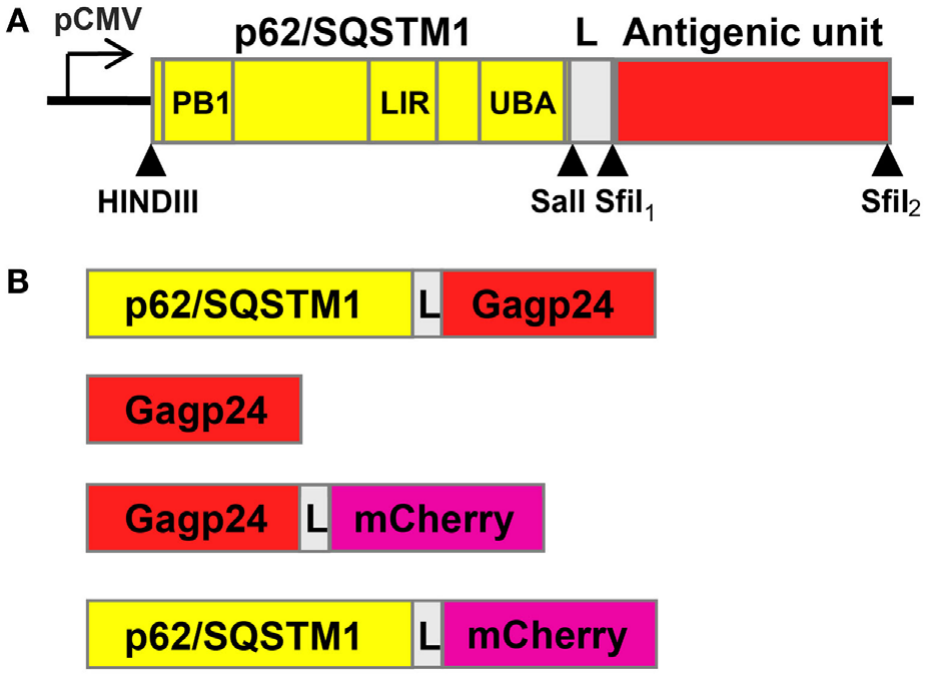
**Schematic drawings of the constructs included in the study**. **(A)** Drawing of the designed p62/SQSTM1-containing vaccine construct. The 3-prime end of DNA of p62 is connected to the antigenic unit *via* a linker region (L) encoding the amino acids RSTGLSGL. The DNA construct was subcloned into the pBudCE4.1 vector, and expression of the gene was driven by human cytomegalovirus immediate-early promoter (pCMV). Arrow heads indicate unique sites for restriction enzymes. Phox/Bem 1p (PB1), LC3-interacting region (LIR), and ubiquitin-associated domain (UBA) encode some of the functional domains of p62. **(B)** Overview of all the constructs that were included in the study, (i) p62–Gagp24 (Gagp24 isolate BH10), (ii) Gagp24, (iii) Gagp24 fused to mCherry, and (iv) p62 fused to mCherry. mCherry was included to have the possibility to track p62 and Gagp24 by live-cell fluorescence microscopy and was subcloned into the antigenic unit. All constructs were subcloned into the pBudCE4.1 vector downstream of pCMV.

We designed a novel vaccine strategy based on the fusion of antigen to the selective autophagy receptor sequestosome 1 (SQSTM1)/p62, which should enable targeted antigen delivery to the autophagy pathway. Our hypothesis was that this approach would result in enhanced antigen presentation on MHC class I and class II molecules and thereby increased generation of antigen-specific CD8^+^ and CD4^+^ T cells, respectively. Therefore, we generated a hybrid vaccine DNA construct in which the gene encoding the antigen was fused with the p62 gene. The p62 protein is known to be selectively sequestered into autophagy by its LIR [microtubule-associated protein 1 light chain 3 (LC3)-interacting region] domain, which binds to LC3, a marker protein of autophagy (47). Other functional domains of p62 include Phox/Bem 1p (PB1), which can polymerize p62 or interact with other PB1-containing proteins (48, 49) and ubiquitin-associated domain (UBA) that binds ubiquitin and polyubiquitin (50–52) (Figure 1A). Aiming not to affect the function of the N-terminal PB1 domain, DNA encoding the test antigen (HIV-1-derived Gagp24) was connected *via* a short linker (L) to the 3-prime end of p62 (Figure 1A). To be able to track p62 and Gagp24 by live microscopy, we subcloned DNA encoding the fluorescent molecule mCherry into the antigenic unit (Figures 1A,B). Gagp24 and mCherry alone were used as controls (Figure 1B).

### Vaccine Antigen Fused to p62 Is Delivered into the Autophagy Pathway

To examine the localization of the various constructs relative to the autophagy pathway, we made use of HEK293 cells stably expressing EGFP fused to the autophagy marker protein LC3 (44). Upon induction of autophagy, EGFP–LC3 will be observed as punctuated or vesicular structures (53–55). Upon fusion of autophagosomes with endosomes and lysosomes, the luminal content is exposed to gradual decrease in pH (56). EGFP is acid-labile with a p*K*a of 6.0 (57). Therefore, to be able to detect EGFP–LC3 throughout the autophagy pathway and hinder degradation of our constructs by pH-regulated enzymes, the samples were treated with NH_4_Cl to block acidification of intracellular vesicles, before analysis by microscopy (Figure S1 in Supplementary Material).

**Figure 2:**
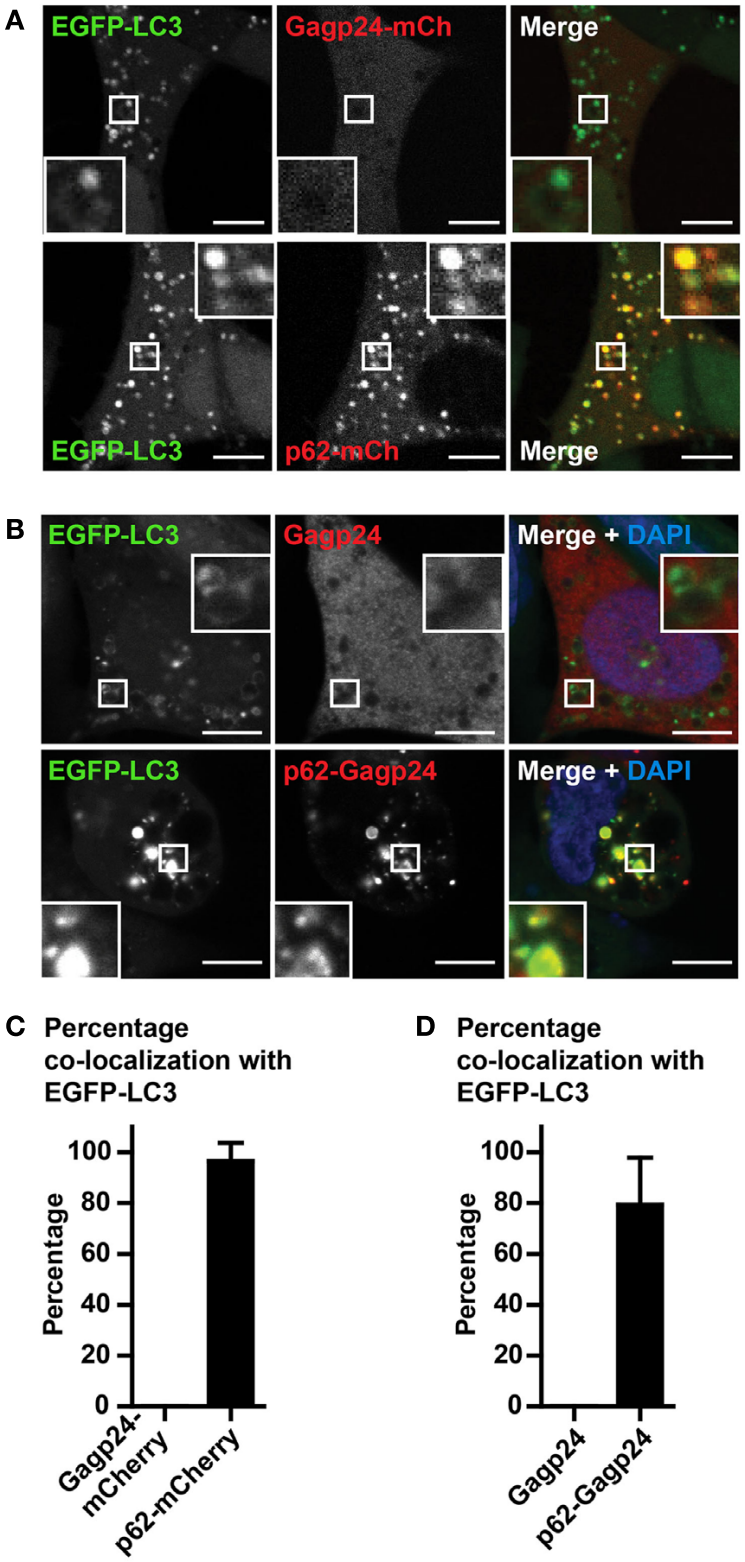
**Gagp24 distributes throughout the cellular cytoplasm**. **(A)** EGFP–LC3^+^ HEK293 cells were transfected with DNA plasmids encoding Gagp24–mCherry or p62–mCherry. After 20 h, 10 mM NH_4_Cl was added to the wells in order to prevent acidification of vesicles and retain the EGFP fluorescent signal. The cells were incubated for additional 24 h before analysis by live-cell confocal microscopy. The corner insets show high magnification of framed areas. Scale bars, 5 μm. Images are representative selections from three independent experiments. **(B)** DNA encoding Gagp24 or p62–Gagp24 was transfected into EGFP–LC3^+^ HEK293 cells. After 20 h, 10 mM NH_4_Cl was added to the wells and incubated for additional 24 h before fixation and staining with anti-Gagp24 antibody. The corner insets show high magnification of framed areas. Scale bars, 10 μm. Images are representative selections from three independent experiments. **(C)** Quantification of double-positive puncta per cell for EGFP–LC3 and Gagp24–mCherry as well as EGFP–LC3 and p62–mCherry in the experiment outlined in **(A)**. Mean values with SEM of three independent experiments are presented. **(D)** Quantification of double-positive puncta for EGFP–LC3 and Gagp24 as well as EGFP–LC3 and p62–Gagp24 per cell in the experiments outlined in **(B)**: 79% of the p62–Gagp24 puncta were positive for LC3 in NH_4_Cl-treated cells. Mean values with SEM of three independent experiments are presented.

**Figure 3:**
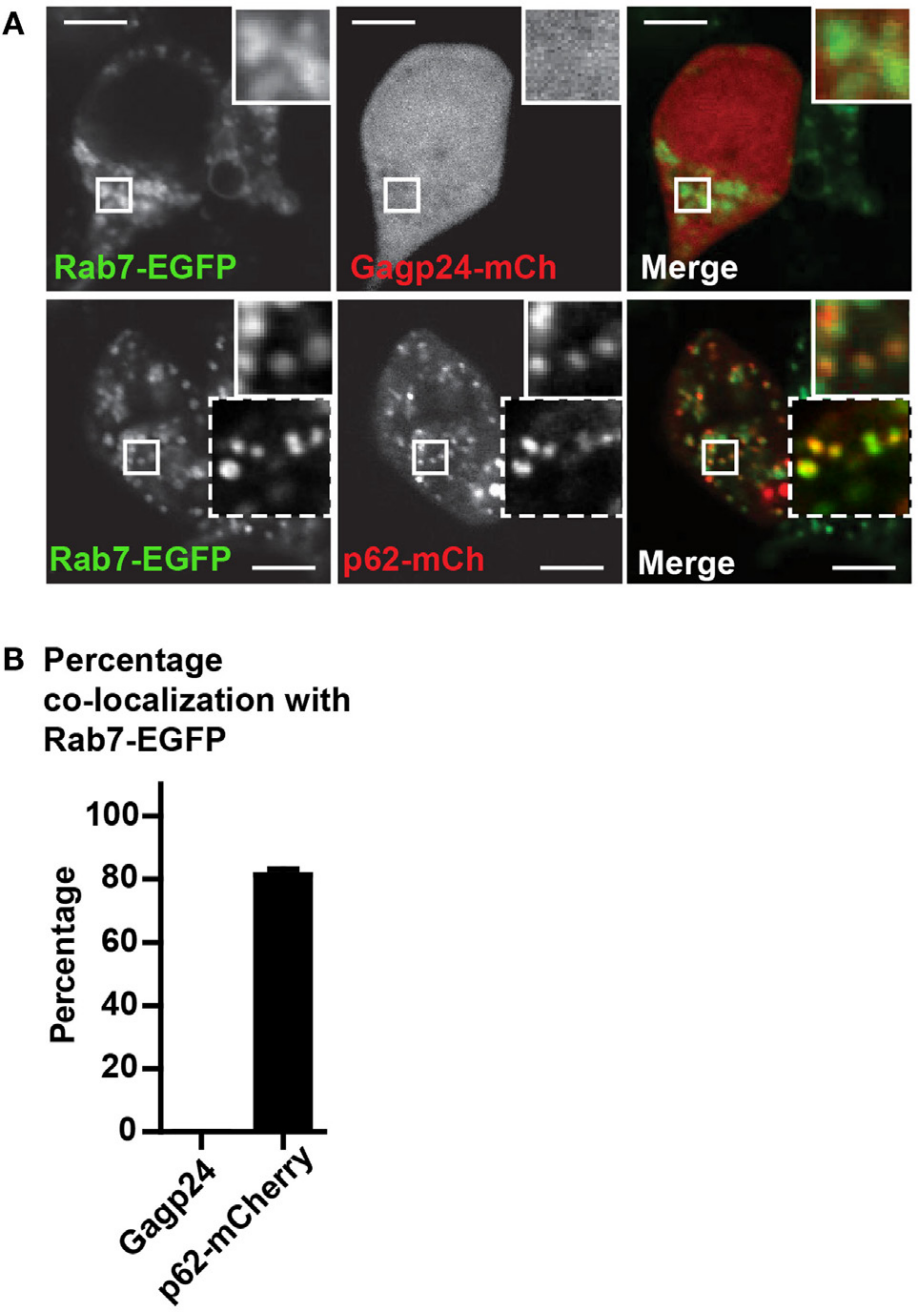
**p62 delivers fused antigen into autophagosomes**. **(A)** HEK293T cells were co-transfected with DNA plasmids encoding Rab7-EGFP and Gagp24–mCherry or p62–mCherry. After 20 h, 10 mM NH_4_Cl was added to the wells to prevent acidification before incubation for additional 24 h and live-cell confocal microscopy. The corner insets show high magnification of framed areas. Scale bars, 10 μm. Images are representative selections from three independent experiments. **(B)** Quantification of double-positive puncta per cell for Rab7-EGFP and Gagp24–mCherry as well as Rab7-EGFP and p62–mCherry in the experiment outlined in **(A)**. Mean values with SEM of three independent experiments are presented.

Human immunodeficiency virus-1 Gag (precursor of Gagp24) has been found in complexes with LC3 (58). As we aimed to evaluate the effect of p62-mediated targeting to autophagy, we needed to exclude that Gagp24 was able to reach this pathway on its own. Therefore, in the first set of experiments, we transfected DNA encoding Gagp24–mCherry into EGFP–LC3^+^ HEK293 cells. Analysis by live-cell confocal microscopy revealed a weak, mostly uniform mCherry signal throughout the cell, suggesting that Gagp24 did not sort into any particular compartment (Figures 2A and 3A, upper panels). In contrast, transfection of DNA encoding p62–mCherry resulted in highly fluorescent mCherry positive puncta in the cytoplasm (Figure 2A, lower panels), consistent with previous reports (33). Whereas there was no enrichment of Gagp24–mCherry in EGFP–LC3^+^ structures, more than 95% of the p62–mCherry puncta co-localized with EGFP–LC3^+^ (Figures 2A,C). Next, we examined whether p62 also could target Gagp24 into the autophagy pathway. EGFP–LC3^+^ HEK293 cells were transfected with DNA plasmids encoding Gagp24 or p62–Gagp24 and cultivated before fixation and immunostaining of Gagp24. In cells transfected with Gagp24 alone, Gagp24 was distributed evenly throughout the cell and was largely excluded from EGFP–LC3^+^ puncta (Figure 2B, upper panels). In contrast, but similar to what we observed for p62–mCherry, p62–Gagp24 accumulated in distinct puncta, which showed overt co-localization with EGFP–LC3 (Figure 2B, lower panels). Quantification of microscopy images revealed that about 80% of the p62–Gagp24 puncta were positive for EGFP–LC3 (Figure 2D). These data suggest that p62 can deliver proteins that are fused to its C-terminus into the autophagy pathway.

Because both LC3 and p62 can be present as aggregates in the cytosol (32, 55), we wanted to confirm that fusion constructs of p62, indeed, was sequestered in autophagic vesicles. For this purpose, we used Rab7-EGFP, a marker of late endosomes, lysosomes as well as mature autophagosomes that have fused with endosomes or lysosomes (i.e., amphisomes and autolysosomes, respectively) (59, 60). HEK293T cells were co-transfected with DNA encoding Rab7-EGFP and Gagp24–mCherry or with Rab7-EGFP and p62–mCherry before live-cell confocal microscopy. There was no co-localization between Rab7-EGFP and Gagp24–mCherry relative to the background [Figure 3A (upper panel) and Figure 3B]. In contrast, more than 80% of the p62–mCherry puncta co-localized with Rab7-EGFP [Figure 3A (lower panel) and Figure 3B]. Thus, the dominant fraction of p62–mCherry co-localized with Rab7, most likely indicating mature autophagic vesicles. Collectively these data suggest that fusion to p62 can be utilized as a means to efficiently deliver vaccine antigens into LC3- and Rab7-positive vesicles, i.e., the autophagy pathway.

### p62 Alters the Level of Vaccine Antigen

**Figure 4:**
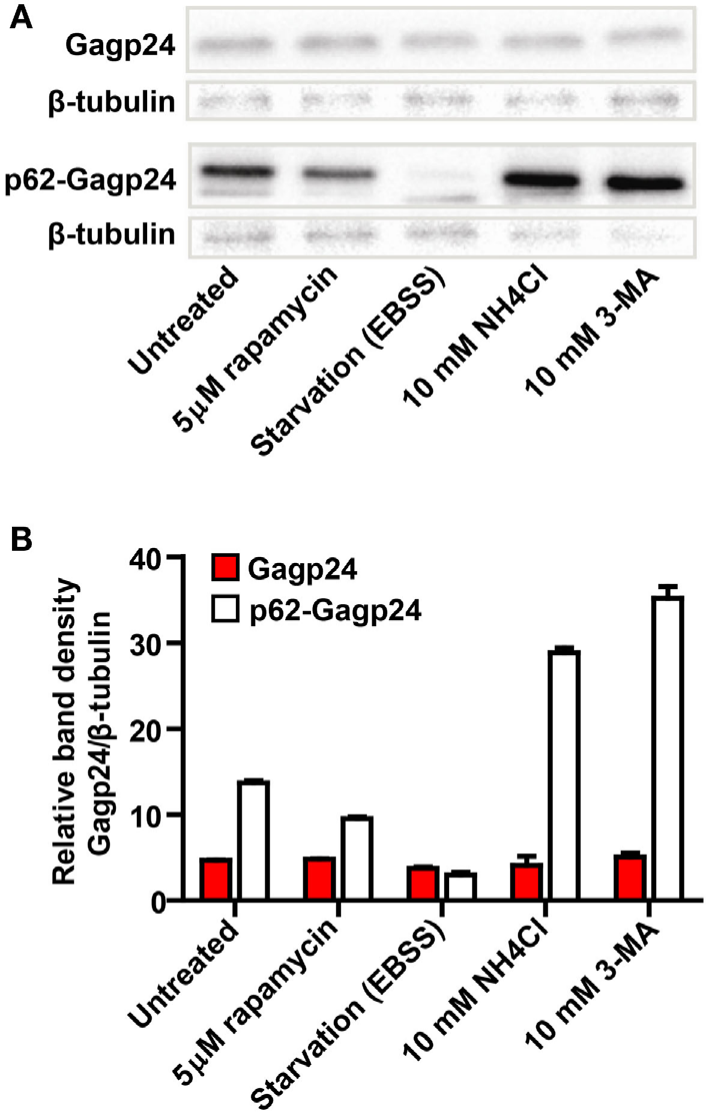
**Modulators of autophagy affect p62–Gagp24 but not Gagp24**. **(A)** DNA plasmids encoding Gagp24 or p62–Gagp24 were transiently transfected into HEK293T cells. At 20 h post-transfection, the cells were exposed for 12 h to treatments that modulate autophagy, 5 μM rapamycin (induces autophagy), amino acid starvation by incubation in Earle’s balanced salt solution (EBSS) (induces autophagy), incubation with 10 mM NH_4_Cl (hinders protein degradation in mature autophagosomes), or 10 mM 3-methyladenine (3-MA) (hinders initiation of autophagy) before making cell lysates. The lysates were analyzed for changes in Gagp24 levels by western blotting, using a monoclonal anti-Gagp24 antibody for detection. Labeling with an anti-β tubulin antibody was used to compensate for loading differences. The experiment was performed twice and the results were similar. **(B)** Results of the experiment outlined above, presented in a histogram, showing band density of Gagp24 normalized to that of β-tubulin. Each column shows mean values with SEM of pooled data from two independent experiments.

To examine whether p62 altered the level and intracellular turnover of Gagp24, HEK293T cells were transfected with DNA encoding Gagp24 or p62–Gagp24 before treatment with inducers or inhibitors of autophagy. Both rapamycin and amino acid deprivation are strong inducers of autophagy. NH_4_Cl prevents endolysosomal acidification and thereby the activity of low pH-dependent proteases derived from endosomes and lysosomes upon autophagosomal maturation. 3-methyladenine (3-MA) is an inhibitor of phosphoinositide 3-kinases including Vps34, which is required for the initiation of autophagy (61, 62). Western blotting of lysed cells revealed that the levels of Gagp24 alone was unaffected by all treatments (Figures 4A,B). In contrast, the level of p62–Gagp24 correlated with the level of autophagy, i.e., the inducers of autophagy (rapamycin or amino acid deprivation) reduced the level of p62–Gagp24, whereas inhibitors (NH_4_Cl or 3-MA) enhanced the level (Figures 4A,B). These data are consistent with the observations made by microscopy, i.e., p62 altered the localization of Gagp24 and targeted antigen to LC3- and Rab7-positive vesicles. Furthermore, the data show that p62–Gagp24 levels were regulated by endolysosomal activities and mechanisms known to control autophagic activity.

### Coupling to p62 Prevents Rapid Degradation of HIV-1 Gagp24 in the Cytosol

**Figure 5:**
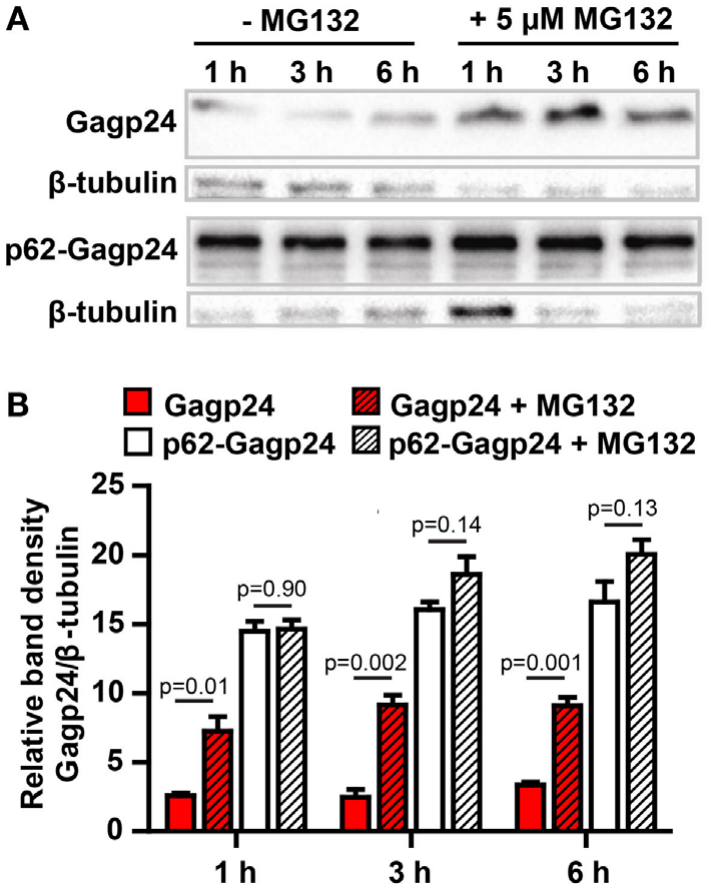
**p62 protects Gagp24 from degradation by the proteasome**. **(A)** DNA plasmids encoding Gagp24 or p62–Gagp24 were transiently transfected into HEK293T cells. At 20 h after transfection, the cells were left untreated or were treated with 5 μM MG132, an inhibitor of the proteasome, for 1, 3, or 6 h before making cell lysates. The lysates were analyzed by western blotting using an anti-Gagp24 antibody. Labeling with an anti-β tubulin antibody was used to compensate for loading differences. The experiment was performed three times, and the results were similar. **(B)** Results of the experiment outlined above, shows band density of Gagp24 normalized to that of β-tubulin. Each column shows mean values with SEM of pooled data from three independent experiments. *P*-values were calculated by two-tailed *t*-test.

We hypothesized that the formation of aggregates and the targeting of antigen into the autophagy pathway should protect the antigen from rapid degradation in the cytosol by the proteasome. In order to test this, half of the transfected HEK293 cells were treated for 1, 3, and 6 h with MG132, an inhibitor of the proteasome (63). The cell lysates were subjected to western blotting and protein was detected using an anti-Gagp24 antibody (Figure 5A). Analysis of the density of the bands revealed that inhibition of the proteasome even for only 1 h led to a significant increase of Gagp24 in the Gagp24-transfected cells (Figure 5B). In contrast, there was no significant increase in the level of p62–Gagp24 in untreated and MG132-treated cells (Figure 5B). These data are consistent with a model where fusion with p62 delivers Gagp24 into the autophagy pathway and thereby prevents it from rapid degradation by the proteasome.

### Antigen Fusion to p62 Results in Increased Generation of Gagp24-Specific T Cells *In Vivo*

**Figure 6:**
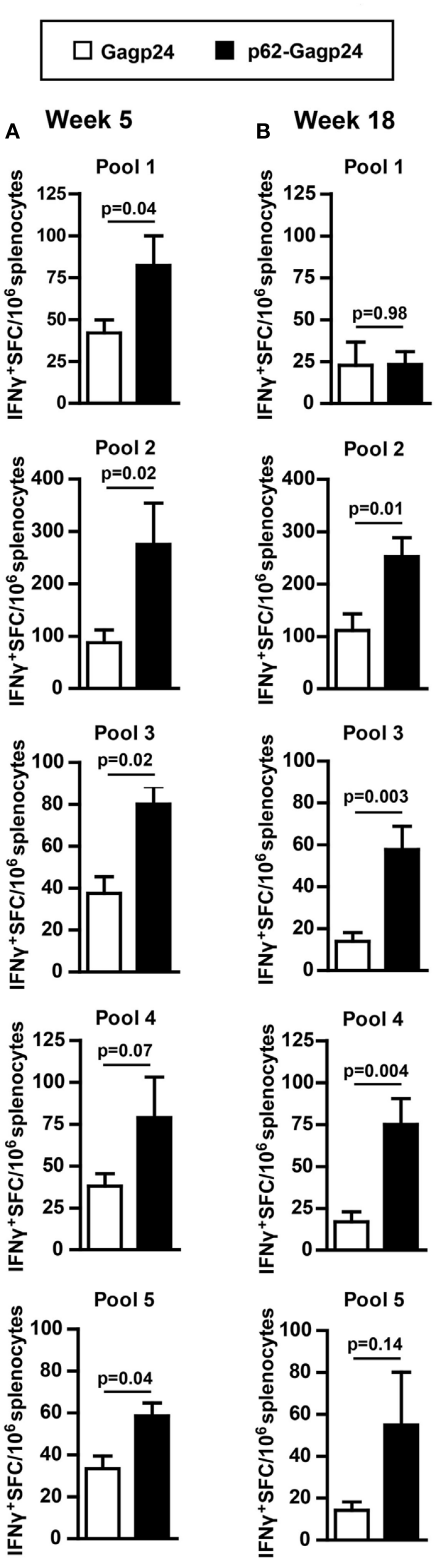
**Fusion of Gagp24 to p62 results in increased number of Gagp24-reactive T cells in mice**. BALB/c mice were immunized once by intradermal injection on the left and right flank with 25 μl of 12.5 μg DNA plasmids encoding Gagp24 or p62–Gagp24 before electroporation. Splenocytes were harvested after 5 **(A)** or 18 **(B)** weeks and subjected to IFNγ-ELISpot for enumeration of IFNγ-producing Gagp24-reactive T cells toward peptides spanning Gagp24. The peptides were 15-mers and were divided in pools 1–5, each pool containing 9–12 peptides. Pool 1 consisted of peptides spanning amino acid 125–183 of Gag polyprotein precursor; pool 2, amino acid 173–231; pool 3, amino acid 221–279, pool 4, amino acid 269–327; and pool 5, amino acid 317–363. Mean values with SEM are shown, week 5: *n* = 3–6 mice/group and week 18: *n* = 7 mice/group. *P*-values were calculated by two-tailed *t*-test.

As the p62 fusion directed the antigen to autophagy and prevented it from rapid degradation, we wanted to examine whether the fusion construct would result in enhanced T cell responses *in vivo*. DNA plasmids encoding Gagp24 or p62–Gagp24 were injected into the dermis of mice before electroporation (which was used to enhance uptake of the DNA). Splenocytes were harvested at different time points after immunization. To evaluate T cell responses, we used a library of 15-amino acid long peptides with an 11-amino acid overlap between sequential peptides covering the full Gagp24 sequence. The peptides were divided into 5 pools each containing 9–12 peptides. Each peptide pool was added to splenocytes, and responding T cells were detected by IFNγ-ELISpot. Because of the length of the peptides, they were expected to bind to MHC class II molecules and be indicative of CD4^+^ T cell responses, and peptides in pools 1 and 3 have previously been shown to stimulate CD4^+^ T cells (45). However, CD8^+^ T cell responses cannot be excluded as peptides being up to 13-amino acids have been shown to restimulate CD8^+^ T cells (64). Moreover, the 15-mers may be trimmed into 8–10 amino acid long peptides, which is the typical length for MHC class I-binding. Immunization with Gagp24 resulted in low number of IFNγ^+^ T cells reactive toward peptide pools 1, 3, 4, and 5 (Figures 6A,B). In comparison to Gagp24 alone, immunization with p62–Gagp24 resulted in a tendency of more IFNγ^+^ T cells toward all peptide pools except from peptide pool 1 (Figures 6A,B). Furthermore, there was a significant increase in IFNγ^+^ T cell responses toward pool 2 and pool 3 peptides at both 5 and 18 weeks (Figures 6A,B). The highest number of IFNγ^+^ T cells was observed toward peptide pool 2, which covered a previously defined Gag197–205 MHC class I-restricted peptide (46).

**Figure 7:**
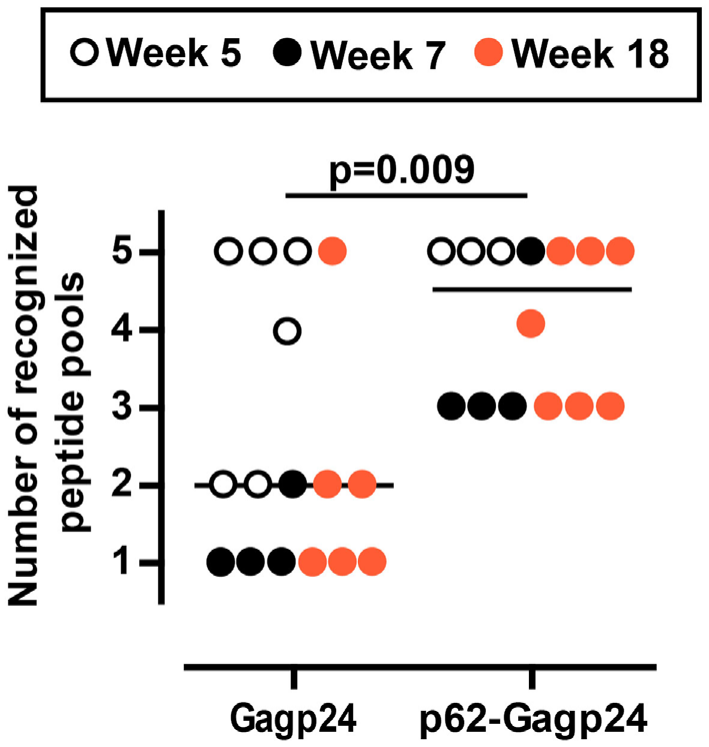
**Mouse responsiveness to number of Gagp24-derived peptide pools**. The graph presents the number of peptide pools that each immunized BALB/c mouse responded to with IFNγ^+^ T cells. The mice were immunized once with an intradermal injection on the left and right flank with 25 μl of 12.5 μg DNA plasmid encoding Gagp24 or p62–Gagp24 before electroporation. Splenocytes were harvested after 5, 7, or 18 weeks and subjected to IFNγ-ELISpot for enumeration of Gagp24-reactive T cell responses toward five different Gagp24-derived peptide pools described in the legend of Figure 6. In the analysis, 20 IFNγ^+^ SFC/106 splenocytes were set as threshold, and only responses toward a given peptide pool that were above the threshold were included. Gagp24, *n* = 16 mice; p62–Gagp24, *n* = 14 mice. The *P*-value was calculated by Mann–Whitney test.

In another set of analysis, we registered the responsiveness of each immunized mice toward a number of peptide pools. An arbitrary value of 20 IFNγ^+^ SFC/10^6^ splenocytes was set as threshold, and only responses above this number toward a given peptide pool were included. In order to include as many mice as possible in the analysis, we plotted data obtained 5, 7, and 18 weeks after immunization and found that the Gagp24 group elicited responses against 1–5 different peptide pools (median value 2) (Figure 7). In the p62–Gag24 group, all mice showed responses toward three or more peptide pools (median value 4.5), which were significantly different from the responses of the Gagp24 group (Figure 7). The analysis shows that in comparison to antigen delivered alone, fusion to p62 resulted in more IFNγ^+^ T cells, which specificity covered 4.5 of the peptide pools. This may imply that fusion to p62 enhances the presentation of antigenic peptides. The peptide repertoire may be identical to that generated following immunization with Gagp24. Alternatively, fusion to p62 promotes the presentation of a more diverse peptide repertoire with the consequent development of a broader pool of HIV-1 Gagp24-specific T cells.

### Fusion to p62 Increased the Number of Gagp24-Reactive CD8^+^ T Cells

**Figure 8:**
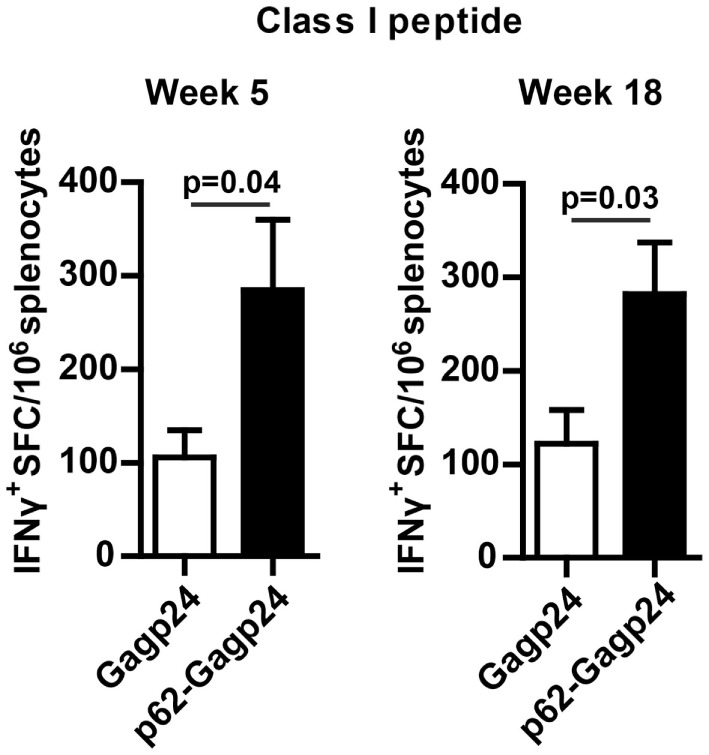
**Fusion of Gagp24 to p62 increased the number of Gagp24-reactive CD8^+^ T cells**. BALB/c mice were immunized once by intradermal injection on the left and right flank of 25 μl of 12.5 μg DNA plasmid encoding Gagp24 or p62–Gagp24 before electroporation. Splenocytes were harvested after 5 or 18 weeks and subjected to IFNγ-ELISpot for enumeration of CD8^+^ T cells, specific for the Gagp24 class I peptide sequence AMQMLKETI (46). Mean values with SEM are presented. Week 5: *n* = 3–6 mice/group and week 18: *n* = 7 mice/group. *P*-values were calculated by two-tailed *t*-test.

Splenocytes examined for their responses toward 15-mer peptides spanning Gagp24 (described in the previous section) were also tested for their recall response toward the MHC class I-restricted peptide Gag197–205 (46). Enumeration of IFNγ^+^ ELISpots showed that p62–Gagp24 induced a significantly higher number of IFNγ-producing CD8^+^ T cells compared to Gagp24 alone (Figure 8). Taken together, analyses of the T cell responses suggest that fusion of antigen to p62 represents an efficient strategy to increase antigen-reactive CD4^+^ as well as the CD8^+^ T cell responses.

## Discussion

In this study, we show that coupling of HIV-1 Gagp24 to p62 promotes the magnitude of the IFNγ^+^ T cell response. To our knowledge, this is the first study that makes use of antigen fusion to p62 to promote vaccine responses. By this strategy, the antigen was efficiently delivered to the autophagy pathway. It is accepted that the autophagy pathway delivers peptides for presentation on MHC class II molecules (13, 21–25). That the pathway also contributes with antigen for presentation on MHC class I molecules is less established (10, 14).

Autophagy can intercept pathogen invasion of the cytoplasm, and both virus and bacteria can be found inside autophagic compartments. Moreover, there is cross talk between autophagy and pattern recognition receptors, and activation of autophagy has been linked to Th1 cytokines (15). Based on these and other findings, we hypothesized that autophagy is involved in antigen delivery to MHC class I molecules. In line with this are the studies that show enhanced CD8^+^ T cell responses following delivery of enriched antigen-containing autophagic vesicles or antigen donor cells with enhanced autophagy (28–31). p62, being a classical autophagic cargo receptor, has been implied in the targeting of pathogens to autophagic degradation. Its activity has also been addressed in delivery of ubiquitinated antigen into autophagosomes and subsequent autophagy engaged cross-presentation of antigen to CD8^+^ T cells (30). Based on these reflections and data, we hypothesized that p62-mediated delivery of antigen into the autophagy pathway may be a viable strategy to promote induction of antigen-specific CD4^+^ and CD8^+^ T cells.

Whereas most studies have fused protein to the N-terminal end of p62, we chose to fuse mCherry and Gagp24 to its C-terminal end. Immunofluorescence microscopy and western blotting showed that p62 retained the ability to be sequestered into the autophagy pathway suggesting that the LIR domain was available for binding to LC3. Moreover, by fusion to the C-terminal end of p62, we anticipated that the PB1 domain would maintain its ability to polymerize with PB1 domain-containing proteins, such as atypical protein C kinases and the protein kinase MEKK3. These interactions are required for NF-κB activation in response to inflammatory signals (such as TNFα and IL-1) mediated by TNF receptor-associated factor 6 (TRAF6) and receptor-interacting protein (RIP) (65–67), which may be beneficial to promote vaccine-induced immunity.

Viral proteins, including the precursor of HIV-1 Gagp24 (Gag), have been reported to associate with the autophagy pathway (58). In our experiments, the protein level of Gagp24 was only affected upon incubation with the proteasome inhibitor, suggesting that Gagp24 was processed by the proteasome and not by autophagy. In contrast, the level of p62–Gagp24 varied dependent on whether autophagy was induced or inhibited, verifying that p62 delivered the antigen into the autophagy pathway. By fusion of Gagp24 to p62, we observed an enhanced number of antigen-specific IFNγ^+^ T cells recognizing the peptide pools 2, 3, 4, and 5 (i.e., peptides covering 80% of the Gagp24 protein sequence). Our data suggest that by fusing antigen to p62 and thereby targeting to autophagy one can increase the magnitude of the IFNγ^+^ T cell response. The results bear similarities with the data of Jin et al., showing that a DNA vaccine based on antigen fusion to LC3 induced, although to a low extent, an antigen-specific CD4^+^ and CD8^+^ T cell response and increased their magnitude (19). There could be several explanations for why fusion to p62, rather than to LC3, appears to be a better strategy for the induction of T cell responses. Constructs of p62 with deletions or mutations in domains that mediate signaling, and in the domain (LIR domain) that mediates targeting to autophagy, may identify the mechanisms behind the enhanced T cell responses.

**Figure 9:**
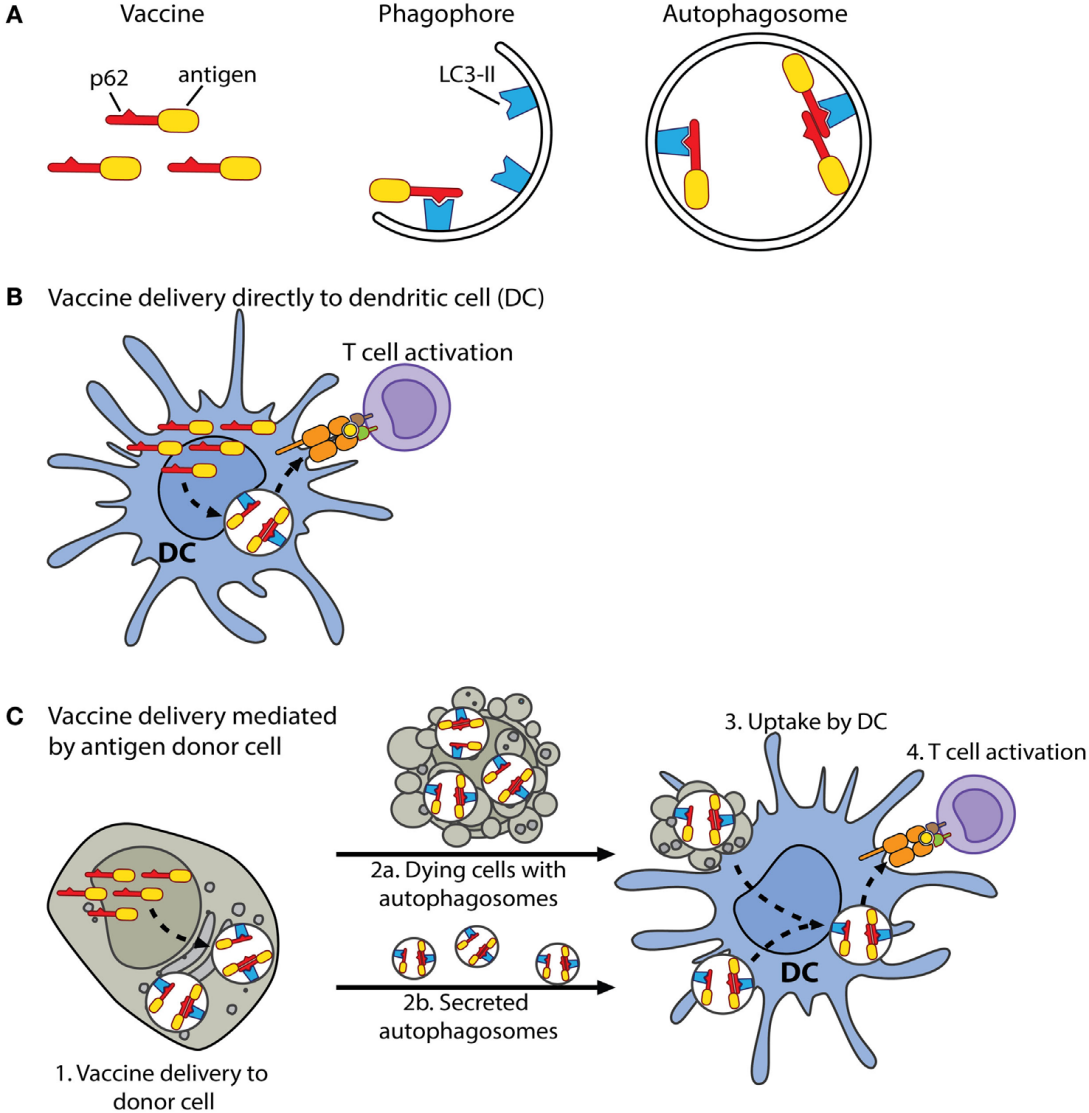
**Schematic drawing of proposed mechanisms of the p62-containing vaccine construct**. **(A)** Antigen is couplet to the C-terminus of p62. Lipidated LC3, LC3-II, is bound to the phagophore, which is a membrane precursor of the autophagosome. The LIR motif of p62 enables interaction with LC3-II. The phagophore expands, eventually seals, and forms an autophagosome. In this manner, p62 can selectively deliver couplet antigen into autophagosomes. **(B)** In mice, when the vaccine is delivered as DNA into the dermis, DCs may be transfected and express the vaccine protein. The vaccine protein is by p62 sequestered in autophagosomes or may first accumulate in DC aggresome-like structures (DALIS) in the cytosol. DALIS are selectively removed from the cytosol by autophagy. Autophagosomes fuse with endocytic compartments, and the antigen is processed in a TAP-dependent or independent manner before its peptides are loaded on MHC class I molecules and transported to the cell surface. **(C)** Alternatively, delivered DNA is taken up by cells, such as fibroblasts and epithelial cells. Expressed vaccine protein is sequestered in autophagosomes of these cells, which now serve as antigen donor cells (1). Dying cells with vaccine-containing autophagosomes are engulfed by DCs (2a and 3). Alternatively, autophagosomes, which may have undergone maturation, are secreted and internalized by cross-presenting DCs (2b and 3). The antigen is processed and loaded on MHC class I molecules *via* pathways that remain to be identified (3). It is well established that autophagy contributes to the pool of cytoplasm-derived antigens displayed on MHC class II molecules (24). Moreover, pathogens, soluble antigens, extracellular vesicles as well as dying cells may be internalized by DCs, and MHC-II-destined peptides are generated by endo/lysosomal proteolysis (9).

Use of an HIV-1-derived MHC class I-restricted peptide (AMQMLKDTI) revealed that fusion to p62 can promote CD8^+^ T cell responses. Because 15-mer peptides may be trimmed and MHC class I molecules have been reported to bind peptides longer than 8–10 amino acids (64), the observed IFNγ^+^ T cell responses towards the peptide pools may also include CD8^+^ T cells. Following delivery of DNA into mice, the p62-containing construct (Figure 9A) may promote T-cell-mediated immunity by different mechanisms, which are not mutually exclusive, (i) DCs may be transfected, and the expressed vaccine protein is by p62 selectively sequestered in autophagosomes (Figures 9A,B). Autophagosomes fuse with endocytic compartments (68), and the antigen may be processed in a TAP-dependent or independent manner before loading on MHC class I molecules. (ii) Cell types other than DCs, such as epithelial cells and fibroblasts, may be transfected and will function as antigen donor cells. The expressed vaccine protein is sequestered in autophagosomes, which may be secreted (69) and internalized by DCs specialized for cross-presentation (Figures 9A,C). Alternatively, dying cells containing autophagosomes with vaccine protein are engulfed by DCs before the antigen is processed and presented on MHC class I molecules to CD8^+^ T cells (Figure 9C). Moreover, it cannot be excluded that the increased number of antigen-specific CD8^+^ T cells is caused by an enhanced number of CD4^+^ T cells available for help. Along these lines, it should be mentioned that Schmid et al. observed some co-localization between LC3 and molecules characteristic for MHC class I loading compartments, although antigen-targeting to autophagy by LC3 did not promote CD8^+^ T cell stimulation *in vitro* (24). Several other reports implicate that autophagy can enhance presentation of MHC class I peptides and priming of CD8^+^ T cells (28–31). Based on these studies, it is reasonable to believe that fusion to p62 may have some direct effect on antigen presentation and activation of CD8^+^ T cells in our experiments.

The number of epitopes that are targeted by CD8^+^ T cells (i.e., the breadth of the response) appears to be important for viral control of HIV-1. *Post hoc* analyses of participants of the HTVN 502 vaccine trial showed that individuals in whom the vaccine had induced T cell responses targeting ≥3 epitopes of Gag, achieved a lower viral load after HIV infection than subjects without Gag responses (71, 72). A broad T cell response may be particularly important in order to target different viral strains and escape mutants and in order to enhance the probability of targeting subdominant epitopes associated with protection against the virus (71, 73). Along these lines, many types of cancers are characterized by point mutations in endogenous proteins and the ability to escape an immune response by immunoediting (74, 75). Thus, the ability to induce a response toward a broad number of epitopes and proteins is expected to be necessary for a vaccine to be efficient against HIV-1 and in therapeutic cancer vaccination.

Mice in the p62–Gagp24 group generated a higher number of IFNγ^+^ T cells specific for more peptide pools than mice in the Gagp24 group. Further studies are needed to determine whether our approach indeed enhances the clonal diversity of the T cell response, and if so, whether that applies to both the CD4^+^ and CD8^+^ T cell responses. Nevertheless, the ability of p62 to divert the antigen away from rapid proteasomal degradation and expose it to another set of proteolytic enzymes does have potential advantages for generation of antigen-specific T cell responses. Exposure to proteasome inhibitors has resulted in enhanced presentation of both dominant and subdominant HIV-1 epitopes (76). Dinter et al. showed that proteases present in the cytosol and the endolysosomal system can differently affect the peptide repertoire (76). Indeed, some peptides were more efficiently generated by enzymes present in the endolysosomal pathway. Moreover, inhibiting rapid degradation of model antigens has been shown to enhance cross-priming of CD8^+^ T cells by the transfer of proteasomal substrate to APCs (70).

In addition to the importance of a broad T cell response, focus has recently been put into the need of inducing T cell responses toward subdominant epitopes. For example, controllers of HIV-1 show enhanced frequency of cytotoxic CD8^+^ T cells specific for subdominant epitopes (71). To induce such T cell responses, the pattern of immunodominance may have to be broken. Therefore, there is a need for the development of novel vaccine strategies. We found that antigen fusion to p62 rescued the antigen from rapid proteasomal degradation and enhanced antigen-specific IFNγ^+^ T cell responses. Our vaccine strategy could be a viable strategy to promote the generation of protective T cell responses.

## Author Contributions

AA and IØ performed the experiments and designed the study; all authors analyzed the data; AA, AC, and IØ wrote the manuscript; and OL and AS contributed in writing the manuscript.

## Conflict of Interest Statement

The TTO office of the University of Oslo and Oslo University Hospital has filed a patent on the vaccine technology, on which AA, BB, and IØ are inventors. This does not alter the authors’ adherence to all the policies on sharing data and materials.
