# Supplementary material for: Coupling of HIV-1 Antigen to the Selective Autophagy Receptor SQSTM1/p62 Promotes T-Cell-Mediated Immunity
Source: Front Immunol. 2016 May 10;7:167. doi: 10.3389/fimmu.2016.00167 (PMC4861724; doi:10.3389/fimmu.2016.00167)
Supplement: Supplementary file 1 [file Presentation_1.pdf]

SUPPLEMENTARY INFORMATION

Coupling of HIV-1 antigen to the selective autophagy receptor SQSTM1/p62 promotes T-cell mediated immunity

Aram Nikolai Andersen, Ole Jørgen Landsverk, Anne Simonsen, Bjarne Bogen, Alexandre Corthay, and Inger Øynebråten

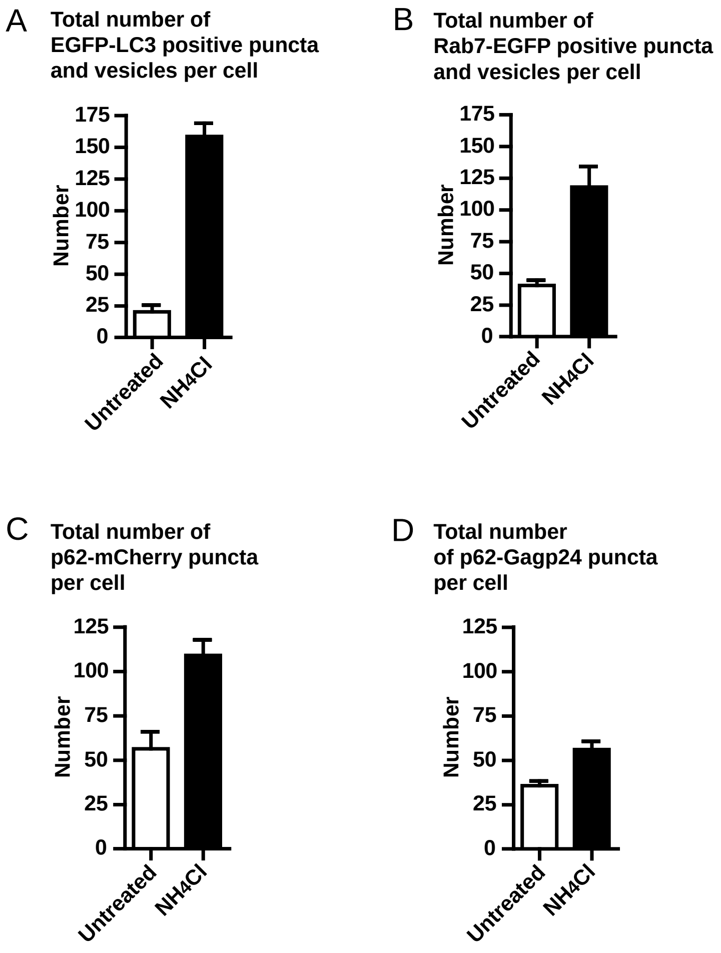

Figure 1S. Quantification of puncta and vesicles in untreated and NH_4Cl-treated cells.
To prevent acidification and degradation of expressed constructs by pH-regulated enzymes, 10 mM NH_4Cl was added to half of the wells 24 h before evaluation of the cells. **(A)** Number of EGFP-LC3 puncta and vesicles per EGFP-LC3$^+$ HEK293 cell transfected with vaccine constructs. **(B)** Number of Rab7-EGFP puncta and vesicles per HEK293T cell co-transfected with vaccine constructs. **(C)** Number of p62-mCherry puncta per transfected HEK293 cell. **(D)** Number of p62-Gagp24 puncta per transfected EGFP-LC3$^+$ HEK-293 cell. **(A-D)** Mean values with SEM of three independent experiments are presented. There was no enrichment of Gagp24-mCherry or Gagp24 in puncta or vesicles.

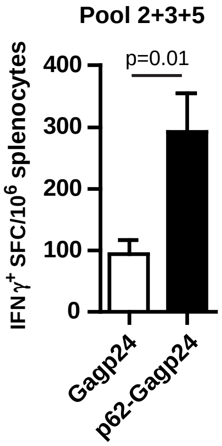

Figure S2. IFNγ-ELISpot of splenocytes re-stimulated with a mixture of Gagp24 derived 15-mer peptides of pool 2, 3, and 5 three weeks after immunization. The C57BL/6 mice were immunized once by intradermal injection on the left and right flank with 25 µl of 12.5 µg DNA plasmid before electroporation. n=6 mice per group. Mean values with SEM are presented, and the p-value was calculated by two-tailed t-test.
